# Prevalence, complications and factors associated with severely elevated blood pressure in patients with hypertension: a cross-sectional study in two hospitals in Yaoundé, Cameroon

**DOI:** 10.11604/pamj.2022.42.20.34146

**Published:** 2022-05-10

**Authors:** Jérôme Boombhi, Joel Gabin Konlack Mekontso, Chris-Nadège Nganou-Gnindjio, Edwige Lea Nzoyem De Hedzo, Guy Loic Nguefang Tchoukeu, Ulrich Igor Mbessoh Kengne, Fabrice Ndzernyuy Dubila, Fabrice Leo Tamhouo Nwabo, Yves Alain Notue, Yannick Kevin Tchiffo Foppa, Alain Patrick Menanga

**Affiliations:** 1 Department of Internal Medicine and Specialties, Faculty of Medicine and Biomedical Sciences, University of Yaoundé I, Yaoundé, Cameroon,; 2 Ministry of Public Health, Cardiology Unit, Yaoundé General Hospital, Yaoundé, Cameroon,; 3 Ministry of Public Health, Djeleng Sub-divisional Hospital, Bafoussam, Cameroon,; 4 Faculty of Medicine and Biomedical Sciences, University of Yaoundé I, Yaoundé, Cameroon,; 5 Ministry of Public Health, Cardiology Unit, Yaoundé Central Hospital, Yaoundé, Cameroon,; 6 Ministry of Public Health, Mengueme Sub-divisional Hospital, Mengueme, Cameroon,; 7 Ministry of Public Health, Messamena Health District Service, Messamena, Cameroon,; 8 Ministry of Public Health, Bangou Sub-divisional Hospital, Bangou, Cameroon,; 9 Ministry of Public Health, Eseka District Hospital, Eseka, Cameroon

**Keywords:** Severe hypertension, Black Africans, cardiological follow-up, Cameroon

## Abstract

**Introduction:**

severely elevated blood pressure significantly increases cardiovascular morbidity and mortality in hypertensive Black patients. The objective of this study was to determine the prevalence, complications and factors associated with severe high blood pressure in hypertensive patients in Yaoundé, Cameroon.

**Methods:**

we conducted a cross-sectional study in the outpatient and cardiology units of two teaching hospitals in Yaoundé. We included consenting hypertensive patients aged over 18 years. We first measured their blood pressure (BP), then we collected their sociodemographic data, cardiovascular risk factors, follow-up data, and ended with a complete physical examination. We performed a regression analysis to assess correlates of severe hypertension.

**Results:**

we included a total of 153 patients with 33 (21.6%) of them having severe hypertension. Among the 33 patients, 16 (48.5%) were male and 17 (51.5%) were female. Their mean age was 60.52 ± 12.83 years. Chronic kidney disease (78.8%), hypertensive retinopathy (69.7%) and left ventricular hypertrophy (48.5%) were the most common complications. On multiple logistic regression analysis, inadequate follow-up was independently associated with severe hypertension (adjusted OR=7.09; 95% CI [2.29-21.9]).

**Conclusion:**

severely elevated BP is common among hypertensive patients in our setting with important physical and economic consequences. Increased patients awareness and improving access to primary care physicians and cardiologists, through health insurance or other means, may be an effective strategy for reducing cardiovascular morbidity and mortality among hypertensive Black patients.

## Introduction

Hypertension is the first cardiovascular risk factor worldwide [1]. In Cameroon, its prevalence is alarming, approaching 30% in some studies, most often evolving insidiously and diagnosed incidentally [2,3]. Some data suggest hypertension is more precocious in Blacks with higher blood pressure (BP) values that are more difficult to control [4]. There is a strong, independent and progressive association between BP levels and the occurrence of adverse cardiac and vascular events [5]. Grade 3 or severe hypertension (systolic BP ≥ 180 mmHg and or diastolic BP ≥ 110 mmHg) leads, without any other cardiovascular risk factors, to a 20-30% risk of cardiovascular disease and is therefore more associated with damage to target organs such as the heart, brain and kidneys [4]. The proportion of severe hypertension among hypertensive patients could be higher in Black Africans than in other populations [6] and severe hypertension remains a common cause of hospitalization and deaths in our setting despite the advent of antihypertensive drugs. Hence, this study aimed to determine the prevalence, complications and factors associated with severe high BP in hypertensive patients in Yaoundé, Cameroon.

## Methods

**Study design and setting:** we conducted a cross-sectional study in the outpatient and cardiology units of the Yaoundé General Hospital and Yaoundé Central Hospital from February to April 2020. These are two referral university hospitals which have many cardiology specialists, a high attendance and receive many patients from all over the country.

**Study population:** sampling was consecutive and all consenting hypertensive patients, aged over 18 years who came for consultation during the study period were included. Our sample size was estimated at 236 participants using the Cochrane formula:


$$N=\{\varepsilon^{2}\times p\times (1-p)\}/l^{2}$$

Where N is the sample size, l=5% is the precision level, ɛ=1.96 is a constant which depends on l, p=19.3% is the prevalence of severe high BP in hypertensive patients previously found by Ngongang *et al*. in Cameroon [3].

**Data collection:** the BP was measured on both arms using a standardized protocol with the participants seated in a quiet room and after at least 15 minutes of rest with validated automated BP machines which were regularly calibrated to avoid erroneous measurements. The mean of two measures performed at least five minutes apart was used for all analyses. We then conducted an anamnesis and used pre-designed and pre-tested questionnaires to collect sociodemographic data, cardiovascular risk factors, follow-up data and ended with a complete physical examination. We used the GIRERD questionnaire to evaluate compliance to treatment [7,8].

**Definitions:** we defined severe high BP as a systolic BP ≥ 180 mmHg and/or diastolic BP ≥ 110 mmHg [9]. High alcohol consumption was defined as men consuming more than 3 glasses of wine per day (2 glasses for women). We considered a patient to be sedentary if he didn´t practice at least 150 minutes of moderate physical exercise per week. A patient was considered properly followed-up if he had at least two office visits with his physician during the past 12 months. The term «associated treatment» essentially referred to lipid lowering drugs and low dose aspirin.

**Statistical analysis:** data analysis was done using SPSS version 23.0 (IBM Corporation, Armonk, NY, USA). Univariate associations were tested with use of the Chi-square test and Fisher´s exact test. We performed a regression analysis to assess independent correlates of severe hypertension. The odds ratios with 95% confidence interval (OR, 95%CI) were used as measure of association.

**Ethical considerations:** our study was reviewed and approved by the Institutional Ethical Clearance Committee of the Faculty of Medicine and Biomedical Sciences of the University of Yaoundé I. A written informed consent was obtained from each participant.

## Results

**General characteristics of the study population:** a total of 153 patients aged 31 to 91 years were included in the study. Severe hypertension was found in 33 (21.6%) patients (Table 1), 16 (48.5%) males and 17 (51.5%) females. Their mean age was 60.52 ± 12.83 years. Age (OR=0.69 [0.30-1.5] 95% CI), sex (OR=1.23 [0.50-2.6] 95% CI) and place of residence (OR=0.53 [0.21-1.31] 95% CI) didn´t influence the distribution of severe hypertension in our study population. Table 2 shows the distribution of the study population according to sociodemographic characteristics. The duration of hypertension ranged from 1 to 20 years with a median duration of 5 years.

**Table 1:** distribution of the study population according to blood pressure levels

Grade of hypertension	Number (n)	Frequency (%)
Normal blood pressure	46	30.1
Grade 1	46	30.1
Grade 2	28	18.3
Grade 3 (severe hypertension)	33	21.6

**Table 2:** distribution of the study population according to sociodemographic characteristics

Variables	Severe hypertension, n (%)	Non-severe hypertension, n (%)	OR [95% CI]
**Sex**			
Male	16 (48.5)	52 (43.3)	1.23 [0.50-2.6]
Female	17 (51.5)	68 (56.7)	
**Age (years)**			
<45	5 (15.1)	12 (10.0)	
45-60	12 (36.4)	39 (32.5)	0.69 [0.30-1.5]
≥60	16 (48.5)	69 (57.5)	
**Residence**			
Urban	24 (72.7)	100 (83.3)	0.53 [0.21-1.31]
Rural	9 (27.3)	20 (16.7)	

**Complications of severe high blood pressure:** chronic kidney disease (78.8%) was the most frequent complication in patients with severe hypertension followed by hypertensive retinopathy (69.7%), left ventricular hypertrophy (LVH) (48.5%) and strokes (27.3%). Table 3 depicts the frequency of complications in patients with severe high BP.

**Table 3:** frequency of complications in patients with severe hypertension

Complications	Number (n)	Frequency (%)
Chronic kidney disease	26	78.8
Hypertensive retinopathy	23	69.7
left ventricular hypertrophy on electrocardiogram	21	63.6
left ventricular hypertrophy on echocardiography	16	48.5
Stroke	9	27.3
Heart failure	7	21.2

**Factors associated with severely elevated blood pressure:** a sedentary lifestyle was the most prevalent cardiovascular risk factor (78.8%) followed by obesity/overweight (75.8%). Only a third of patients with severe hypertension were strictly following a low salt diet, i.e < 5 grams/day. However, these risk factors were equally distributed in both groups (p>0.05). On multivariate analysis, inadequate follow-up was independently associated with severe hypertension (adjusted OR=7.09; 95% CI, 2.29 to 21.9) as depicted in Table 4.

**Table 4:** factors associated with severely elevated blood pressure

Variables	Severe hypertension, n (%)	Non-severe hypertension, n (%)	OR [95% CI]	Aor [95% CI]
**Tobacco**				
Yes	7 (21.2)	8 (6.7)	3.76 [1.13-9.70]	2.7 [0.46-15.18]
No	26 (78.8)	112 (93.3)		
**Alcoholism**				
Yes	18 (54.5)	34 (28.3)	3.03 [1.37-6.70]	2.3 [0.74-7.39]
No	15 (45.5)	86 (71.7)		
**Dyslipidaemia (N=113)**				
Yes	14 (51.9)	20 (23.2)	3.55 [1.43-8.70]	3.15 [0.93-10.5]
No	13 (48.1)	66 (76.8)		
**Proper follow-up (N=142)**				
No	13 (50.0)	19 (16.4)	5.10 [2.82-14.90]	7.09 [2.29-21.9]
Yes	13 (50.0)	97 (83.6)		
**Quality of practitioner (N=110)**				
Cardiologist	10 (77.0)	94 (96.9)	0.10 [0.05-0.59]	0.07 [0.01-1.08]
General practitioner	3 (23.0)	3 (3.1)		
**Compliance to treatment (N=142)**				
Good	0	15 (12.9)	2.62 [1.10-6.20]	1.86 [0.82-6.8]
Average	13 (50.0)	69 (59.5)		
Poor	13 (50.0)	32 (27.6)		
Associated treatment				
Yes	2 (6.0)	29 (24.2)	0.20 [0.04-0.89]	0.60 [0.01-1.35]
No	31 (94.0)	91 (75.8)		
**Left ventricular hypertrophy on electrocardiogram (N=134)**				
	21 (70)	47 (45.2)	2.8 [1.18-6.76]	1.52 [0.49-4.72]
	9 (30)	57 (54.8)		

## Discussion

The objective of this study was to determine the prevalence, complications and factors associated with severely elevated BP in a population of hypertensive Black patients. The prevalence is pretty high and the complications are numerous. Most factors associated with severe rise in BP values are modifiable. These can be targeted for secondary prevention. There is a statement that hypertension is an aging disorder [10]. This statement is verified in our study as 48.5% of our study population was aged over 60 years. Aging is associated with gradual fragmentation and loss of elastin fibers and accumulation of collagen fibers in the tunica media of large arteries causing arterial stiffness. Many authors working on severe hypertension in Blacks and even Caucasians found a mean age greater than 50 years [3,6,11]. We found a higher proportion of women in our survey (sex ratio = 0.94). Men are generally at greater risk for hypertension and cardiovascular diseases than are age-matched, premenopausal women. After menopause, which occurs around 50 years, BP increases in women to levels even higher than in men [12]. Similar sex ratios were found in other studies [3,6,11].

Previous studies suggested that severe high BP was more frequent in Black hypertensive patients. One out of five hypertensive patients in our study had severely elevated BP values. Some authors emphasized on psychological stress and socioeconomic factors to explain the excess and severity of hypertension in Blacks. Others focused on biological and pathophysiological reasons. Because of their genetic make-up, Black patients tend to retain more salt and water and develop a volume-dependent type of hypertension [13,14]. These explanations could justify the higher prevalences found in Cameroon (19.3% in 2016) [3] and Ivory Coast (70% in 2001) [6] as compared to the much lower ones found in Pakistan (16.3% in 2014) [15] and in the United States (11.4% in 2018) [16].

Complications of severe hypertension were frequent in our study, led by Chronic Kidney Disease (CKD) (78.8%). Kidney involvement is common in African studies [3,17-19]. Chronic kidney disease occurs due to the deleterious effects that increased BP has on kidney vasculature. Long-term, uncontrolled, high BP leads to high intraglomerular pressure, glomerular injury and scarring impairing glomerular filtration [20], thus patients with hypertension should have a urine dipstick done and serum creatinine measured regularly. Hypertensive retinopathy was also frequent (69.7%). This corroborates the statement that hypertensive retinopathy is much more prevalent in Black Africans than in Caucasians [21]. Hypertensive retinopathies could regress with adequate BP control, however, no study showed that this regression leads to reduced cardiovascular morbidity and mortality [21]. Almost half patients with severely elevated BP values in our study had concentric LVH confirmed on echocardiography. Left ventricular hypertrophy is a pejorative marker for the occurrence of complications. Not only it is the witness of long-standing uncontrolled hypertension, but it is also a prognostic factor for sudden death [22-24]; hence the importance of having echocardiography done regularly. Stroke was the next complication. There is a close relationship between hypertension and stroke in Blacks. Watila *et al*. [25] as well as Kramoh *et al*. [26] found hypertension as the first risk factor for stroke. Similarly, Sonou *et al*. [27] working on the absolute cardiovascular risk and complications of arterial hypertension in Cotonou, Benin, found that strokes were much more frequent when the systolic BP rose above 170 mmHg. Cardiovascular risk factors were frequent in our study population. Three quarters of patients with severe hypertension were obese or overweight. This result is well above the 23.5% prevalence of obesity found in the Cameroon (both normotensives and hypertensive) in 2014 [2] and suggests that most severe hypertensive patients are found among obese. In addition, this result can be explained by the fact patients are not well aware of the effect of obesity and other risk factors on their BP, but also by poor compliance with dietary measures imposed by hypertension. This unawareness and poor compliance further explains why only a third of patients in our study were strictly following a low salt diet.

Tobacco smoking, high alcohol consumption and dyslipidemia were significantly associated with severe hypertension on univariate analysis. Smoking (nicotine) transiently increases sympathetic autonomic nervous system output but also has a long term effect on lipid metabolism and insulin resistance, involved in atheromatous disease. It also accelerates arterial aging, leading to increased stiffness which is seen in chronic hypertension. Smoking is often associated with increased alcohol consumption, all of which contribute to poor BP control and increased cardiovascular risk [28-30]. Numerous studies have demonstrated a strong relationship between alcohol consumption and hypertension. The mechanisms are multiple: direct action of alcohol on smooth muscle cells, indirect action by stimulating hormones involved in water and sodium regulation and arteriolar vasoconstriction [31,32]. Dyslipidemias have a direct effect on the atheromatous process, which favors the development of renal artery stenosis, an aggravating cause of hypertension [33,34]. However, multivariate analysis failed to show an association between any of these factors and severe hypertension probably because of our smaller than calculated study population.

Unlike Shea´s results [11], a poor compliance to antihypertensive treatment was only marginally associated with severe hypertension (adjusted OR=1.86 [0.82-6.8] 95% CI) in our study probably because of the small size of our population. Compliance with treatment is widely recognized as a key issue in achieving BP control. The cost of antihypertensive medication and dosing frequency could be a few examples contributing to non-compliance and resulting in poorly controlled hypertension. Lack of regular BP monitoring and knowledge of what is the normal BP could also contribute.

Shea *et al*. in the United States found that severe, uncontrolled hypertension was significantly more common among patients who had no primary care physician (adjusted OR=3.5 [1.6-7.7]) and among those who didn´t strictly adhere to their antihypertensive treatment (adjusted OR=1.9 [1.4-2.5]) [11]. An inadequate follow-up was the single factor independently associated with severe hypertension (adjusted OR=7.09 [2.29-21.9]) in our study. This is probably because patients who are well followed are reminded by their primary care physicians to adopt healthier lifestyles, to take their treatments as prescribed and carry-out routine follow-up exams. The poor follow-up in our study could be explained in most cases by lack of financial means and health insurance. Of the 153 patients in our study, only 3 had a health insurance. On top of that, most patients with hypertension are asymptomatic or experiencing only mild nonspecific symptoms like headache and fatigue and will not take the disease seriously until they experience more severe symptoms or complications.

This study took place in the early beginnings of the COVID-19 pandemic in Cameroon. Many patients we approached didn´t consent to participate in our study. Further, the short period of recruitment contributed to the small sample size we had. On top of that, many files were incomplete. Patients were reluctant to do laboratory exams, claiming financial reasons.

## Conclusion

The prevalence of severely elevated BP is high among hypertensive patients in our setting and its consequences are physically and economically important. Tremendous efforts should be made to educate hypertensive patients and encourage them to get involved in the long-term management of their disease. Further, improving access to primary care physicians and cardiologists, through health insurance or other means, may be an effective strategy for reducing cardiovascular morbidity and mortality among hypertensive Black people.

### What is known about this topic

- Severely elevated blood pressure significantly increases cardiovascular morbidity and mortality in hypertensive patients and could be more prevalent in Blacks.

### What this study adds

- The results of this study suggest that educating hypertensive patients and improving their access to primary care providers will help address the high morbidity and mortality of this condition.

## References

[1] Kearney PM, Whelton M, Reynolds K, Muntner P, Whelton PK, He J (2005). Global burden of hypertension: analysis of worldwide data. The lancet.

[2] Kingue S, Ngoe CN, Menanga AP, Jingi AM, Noubiap JJN, Fesuh B (2015). Prevalence and risk factors of hypertension in urban areas of Cameroon: a nationwide population-based cross-sectional study. J Clin Hypertens.

[3] Ngongang Ouankou C, Chendjou Kapi LO, Azabji Kenfack M, Nansseu JR, Mfeukeu-Kuate L, Ouankou MD (2019). Severe high blood pressure recently diagnosed in an urban milieu from Subsahelian Africa: epidemiologic, clinical, therapeutic and evolutionary aspects. Ann Cardiol Angeiol (Paris).

[4] Muntner P, Lewis CE, Diaz KM, Carson AP, Kim Y, Calhoun D (2015). Racial differences in abnormal ambulatory blood pressure monitoring measures: results from the Coronary Artery Risk Development in Young Adults (CARDIA) Study. Am J Hypertens.

[5] Williams B, Mancia G, Spiering W, Agabiti Rosei E, Azizi M, Burnier M (2018). 2018 ESC/ESH Guidelines for the management of arterial hypertension: the task force for the management of arterial hypertension of the European Society of Cardiology (ESC) and the European Society of Hypertension (ESH). Eur Heart J.

[6] Adoubi KA (2006). Aspects épidémiologiques cliniques et thérapeutiques de l'hypertension artérielle à Bouaké. BVS.

[7] Girerd X, Hanon O, Anagnostopoulos K, Ciupek C, Mourad JJ, Consoli S (2001). Assessment of antihypertensive compliance using a self-administered questionnaire: development and use in a hypertension clinic. Presse Medicale Paris Fr.

[8] Girerd X, Radauceanu A, Achard JM, Fourcade J, Tournier B, Brillet G (2001). Evaluation of patient compliance among hypertensive patients treated by specialists. Arch Mal Coeur Vaiss.

[9] Benkhedda DS (2001). Les nouvelles recommandations de l´OMS Société internationale de l´HTA 1999 critiques et controverses. Médecine Maghreb.

[10] Sun Z (2015). Aging, arterial stiffness, and hypertension. Hypertension.

[11] Shea S, Misra D, Ehrlich MH, Field L, Francis CK (1992). Predisposing factors for severe, uncontrolled hypertension in an inner-city minority population. N Engl J Med.

[12] Reckelhoff JF (2001). Gender differences in the regulation of blood pressure. Hypertension.

[13] Saunders E (1987). Hypertension in Blacks. Med Clin North Am.

[14] Spence JD, Rayner BL (2018). Hypertension in Blacks. Hypertension.

[15] Almas A, Ghouse A, Iftikhar AR, Khursheed M (2014). Hypertensive crisis, burden, management, and outcome at a tertiary care center in Karachi. Int J Chronic Dis.

[16] Waldron FA, Benenson I, Jones-Dillon SA, Zinzuwadia SN, Adeboye AM, Eris E (2019). Prevalence and risk factors for hypertensive crisis in a predominantly African American inner-city community. Blood Press.

[17] Renambot J, Adoh MA, Ekra A, Assamoi MO, Bertrand E (1989). Les super-hypertensions artérielles (poussées hypertensives> 250/150). Cardiol Trop.

[18] Diallo AD, Ticolat R, Adom AH, Niamkey EK, Beda BY (1998). Étude de la mortalité et des facteurs de léthalité dans l´hypertension artérielle de l´adulte noir Africain. Médecine Afr Noire.

[19] Yameogo RA, Mandi DG, Yameogo NV, Millogo GRC, Kologo KJ, Toguyeni BJY (2014). La super hypertension artérielle en milieu cardiologique au Burkina Faso. Annales de cardiologie et d´angéiologie. Ann Cardiol Angeiol (Paris).

[20] Gargiulo R, Suhail F, Lerma EV (2015). Hypertension and chronic kidney disease. Dis Mon.

[21] Wong TY, Mitchell P (2004). Hypertensive retinopathy. N Engl J Med.

[22] Yaméogo NV, Kagambèga LJ, Millogo RCG, Kologo KJ, Yaméogo AA, Mandi GD (2013). Facteurs associés à un mauvais contrôle de la pression artérielle chez les hypertendus noirs africains: étude transversale de 456 hypertendus burkinabé. Ann Cardiol Angéiologie.

[23] Samy-Modeliar S, De Cagny B, Fournier A, Slama M (2003). Hypertension artérielle maligne. Réanimation.

[24] Zampaglione B, Pascale C, Marchisio M, Cavallo-Perin P (1996). Hypertensive urgencies and emergencies: prevalence and clinical presentation. Hypertension.

[25] Watila MM, Ibrahim A, Balarabe SA, Gezawa ID, Bakki B, Tahir A (2012). Risk factor profile among Black stroke patients in Northeastern Nigeria. J Neurosci Behav Health.

[26] Kramoh KE, Aké-Traboulsi E, Konin C, N´goran Y, Coulibaly I, Adoubi A (2012). Management of hypertension in the elderly patient at abidjan cardiology institute (ivory coast). Int J Hypertens.

[27] Sonou DA, Lemone H, Adjagba P, Codjo L, Hounkponou M, Houehanou-Sonou C (2017). Etude du risque cardiovasculaire absolu et des complications de l´hypertension artérielle dans une population de patients hypertendus à Cotonou. J Société Biol Clin Bénin.

[28] Cavusoglu Y, Timuralp B, Us T, Akgün Y, Kudaiberdieva G, Gorenek B (2004). Cigarette smoking increases plasma concentrations of vascular cell adhesion molecule-1 in patients with coronary artery disease. Angiology.

[29] Bowman TS, Gaziano JM, Buring JE, Sesso HD (2007). A prospective study of cigarette smoking and risk of incident hypertension in Women. J Am Coll Cardiol.

[30] Halperin RO, Michael Gaziano J, Sesso HD (2008). Smoking and the risk of incident hypertension in middle-aged and older men. Am J Hypertens.

[31] Juillière Y, Gillet C (1996). Alcool et hypertension artérielle. Alcoologie Paris.

[32] Tomson J, Lip GY (2006). Alcohol and hypertension: an old relationship revisited. Alcohol.

[33] Alberti K, Eckel RH, Grundy SM, Zimmet PZ, Cleeman JI, Donato KA (2009). Harmonizing the metabolic syndrome. Circulation.

[34] Chatterjee A, Harris SB, Leiter LA, Fitchett DH, Teoh H, Bhattacharyya OK (2012). Prise en charge des risques cardiométaboliques en soins primaires: résumé de la déclaration consensuelle de 2011. Can Fam Physician.

